# Low-Volume Squat Jump Training Improves Functional Performance Independent of Myofibre Changes in Inactive Young Male Individuals

**DOI:** 10.3390/healthcare10071217

**Published:** 2022-06-29

**Authors:** Ashwin Wayne Isaacs, Kathryn Helen Myburgh, Filippo Macaluso

**Affiliations:** 1 Department of Human Biology, University of Cape Town, Cape Town 7925, South Africa; ash.isaacs@uct.ac.za; 2 Department of Physiological Sciences, Stellenbosch University, Stellenbosch 7600, South Africa; khm@sun.ac.za; 3 SMART Engineering Solutions & Technologies (SMARTEST) Research Center, eCampus University, 22060 Novedrate, CO, Italy

**Keywords:** exercise intervention, peak power, maximal squat jump height, cross-sectional area, muscle fibre type, performance indicators

## Abstract

An investigation into the histological changes in skeletal muscle fibres and jump performance indicators after 8 weeks of plyometric squat jump training was conducted. Healthy inactive participants (n = 13; age: 21.5 ± 1.7 year.; height: 173.6 ± 10.7 cm; weight: 68.5 ± 18.4 kg; BMI 22.4 ± 3.8 kg/m^2^) were recruited, where eight participants completed plyometric squat jump training and five control participants refrained from performing any jumping activities. Blood samples, vastus lateralis muscle biopsies and functional testing (peak and average power, peak and average velocity, maximal jump height) were collected/recorded 10 days prior to and 3 days after the training/rest period. Participants completed 1644 squat jumps over an 8-week training period of 24 sessions with a progressive increase in the number of squat jumps. The trained group significantly increased their jumping average and peak power (mean increases in average power: 16.7 ± 1.2% and peak power: 8.2% ± 0.1) and velocity (mean increases in average velocity: 13.7 ± 0.1% and peak velocity: 5.2% ± 0.03), resulting in a 25% improvement in vertical jump height. No muscle morphological changes in terms of the cross-sectional area (CSA) or muscle-fibre-type transition were observed after the plyometric training. Improvements in the functional performance indicators following training may more likely be explained by sarcomere ultrastructural adaptation, which did not directly affect myosin heavy chain or CSA.

## 1. Introduction

The COVID-19 pandemic resulted in most countries entering a strict lockdown with outdoor activities banned for weeks to months, which posed a significant challenge for staying physically active [1]. This represents a concern, as just days of inactivity can induce muscle loss, loss of muscle strength and power, muscle fibre denervation and neuromuscular damage [2]. A study recruiting Spanish university students found that young adults reduced both their moderate and vigorous physical activity during confinement, while their sedentary time significantly increased. Furthermore, women adapted better to the confinement in terms of physical activity than men, with men being the most sedentary [1,3].

Squat jumps are included in many different exercise protocols for untrained and elite athletes and contribute to vertical jump height performance, which is vital for achieving success in many sporting events [4,5]. Plyometric jump training results in several adaptation responses aimed at improving muscle strength, power and physical performance by activating additional motor units, increasing the reflex potentiation and producing elastic property adaptations to muscle and connective tissue [6].

Previous reports suggested that neural adaptation accounts for improved muscle performance gains rather than muscle architectural change resulting from plyometric exercise training [7]. This was proposed to occur due to neuromuscular changes in the elasticity of muscle, stretch reflex and Golgi tendon organs [8]. However, neuromuscular measures were not included as part of the outcomes of this study. Potteiger et al. speculated that muscle performance improvements observed 8 weeks after plyometric training could be due to an increase in the muscle CSA and the improved recruitment of motor units [9]. Further studies support this proposal, accrediting improvements in jump performance to enhanced contractile strength in a muscle with the addition of motor units and muscle action of agonist and antagonist muscles [8,10]. However, adaptations in the muscle itself cannot be excluded. A change in muscle fibre type following stretch-shortening cycle (SSC) training was shown in animal model studies [11,12]. Limited human studies evaluated fibre-type transitions in response to plyometric jump training alone and these study results contrast with each other. Studies conducted by Kyröläinen et al. and Potteiger et al. revealed no significant muscle fibre transitions in both the gastrocnemius and vastus lateralis muscles 15 and 8 weeks after plyometric training, respectively [5,9]. However, Malisoux et al. reported significant increases in type IIa muscle fibres in response to 8 weeks of different SSC training [13]. They further demonstrated that plyometric training increased the type I and type II fibre CSAs in sports-active individuals not engaging in plyometric jumping [13]. It is important to note that these subjects trained for at least 3 h per week practicing activities such as soccer, hockey, swimming, cycling, judo and gymnastics. In contrast, Vissing et al. observed no increase in the muscle fibre CSA of untrained individuals but found that the whole-muscle CSA increased significantly [6]. This finding could be due to different techniques rather than indicating hyperplasia. A combination of plyometric training coupled with strength training seemed to result in the greatest gains in CSA, as demonstrated by Perez-Gomez et al., who reported significant muscle hypertrophy increases (+4.3%) [14]. Herrero et al. also reported significant increases in the CSA (7.1%) when plyometric training was combined with electro-myostimulation over 4 weeks of training compared with plyometric training alone [15].

Muscle strength and power enhancements may thus be attributed to neural and muscular changes, as demonstrated by authors studying the mechanisms behind these systems in the same study [5,13,16]. Evidence of improved leg extensor power coupled with significant increases in the type I and type II CSAs of vastus lateralis muscle fibres were provided by Potteiger et al. [9]. Previously, we showed that a bout of plyometric jumping in untrained individuals mainly damages type II muscle fibres [17]. In particular, the most damaged fibres were the type IIx (14.3%), followed by the type IIa (10.3%) and then type I (7.6%). Since studies investigating the effect of muscle-fibre-type-specific adaptations following specific plyometric jump type training are lacking, we hypothesised that low-volume plyometric squat jump training in inactive individuals would induce a skeletal muscle fibre transition, increasing type II muscle fibres and result in fibre-type-specific hypertrophy. Studies that showed increases in the skeletal muscle CSA following plyometric training mostly included a variety of jumping exercises, while their study participants had a history of training that included aerobic and resistance training. It is thus important to investigate skeletal muscle histological changes in inactive subjects who are at risk of injury and exertional rhabdomyolysis when performing plyometric jump training [18,19,20]. Moreover, it is important to discern the muscle changes of specific plyometric jump types, which form part of whole-body high-intensity interval training exercises. The current study provided insight into the changes that occur in the first phase of adaptation during a low-volume specific squat jump training protocol in inactive individuals since squat jumps form part of high-intensity interval training [21].

After being sedentary due to a pandemic, young adult individuals are seeking effective and safe training regimens to regain muscle mass and strength. Plyometric jumping imparts strenuous force loads on an individual’s eccentric contracting active muscle groups [22] and thereby improves muscle power and strength; it is thus important to introduce this type of training with care.

## 2. Materials and Methods

### 2.1. Study Design

**Figure 1:**
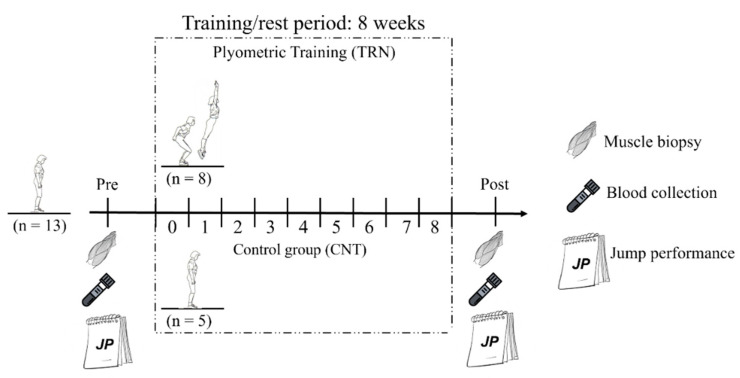
Study design.

Ethical clearance for the study (reference no. N09/05/164) was obtained from the Research Committee of the University of Stellenbosch. Study participants took part in 8 weeks of plyometric jump training (TRN group) or rest (CNT group). A medical doctor collected muscle biopsies from the study participants’ vastus lateralis muscles of legs selected at random at baseline (PRE) and after (POST) the jump-training/rest period (10 days prior and 3 days post-jump-training, respectively). Each participant’s muscle biopsy was obtained from the same leg. Jump performance measures and blood samples were documented and collected 10 days prior and 3 days following the training/rest period. The recruited volunteers participated in a more sophisticated training protocol (previously published [23]) involving an acute bout of plyometric jumps 4 days after the PRE and the POST muscle biopsies that did not affect the disclosed results (Figure 1).

### 2.2. Subjects

Thirteen inactive healthy male participants (age: 21.5 ± 1.7 year.; height: 173.6 ± 10.7 cm; weight: 68.5 ± 18.4 kg; body mass index 22.4 ± 3.8 kg/m^2^) were enrolled for the study. The participants were defined as inactive because they partook in no to little physical activity as shown by the non-achievement of physical activity guidelines [24]. The participants provided written informed consent after being made aware of the experimental procedures and associated risks involved in the study. The participants had no medical conditions or any lower limb injury 6 months prior to the commencement of the study. Participants were omitted from the study if they were chronically treated with any corticosteroid medications (inhaled forms included). The sedentary study participants were reminded to refrain from unaccustomed or robust physical activity for two weeks prior to the commencement and the study duration to allow the participants to recover from the muscle biopsy and to make sure that muscle damage markers in the blood were at baseline levels. Moreover, the participants were encouraged to continue their usual dietary practices and abstain from anti-inflammatory medication during the experimental period.

### 2.3. Plyometric Control and Training Groups

Inactive participants with no training background were allocated to the CNT (n = 5; age, 21.4 ± 1.7 year.; height, 169.3 ± 10.2 cm; weight, 63.0 ± 6.8 kg) or TRN (n = 8; age, 21.4 ± 1.3; height, 176.6 ± 13.0; weight, 75.4 ± 27.9) group at random (Table 1). During the first two weeks, the TRN group performed 5 sets of 5 maximal squat jumps, followed by 7 sets of 7 maximal squat jumps over the next two weeks and 10 sets of 10 maximal squat jumps in the remaining four weeks, while being allowed 1 min rest intervals between sets. All participants reported to the testing site at the same time of day and trained three times per week. The trained group completed 1644 plyometric squat jumps over the 8 weeks training period consisting of 24 sessions, with the number of jumps increasing progressively, as described above. Prior to the commencement of training, the participants completed a 10 min warm-up (5 min of backward and forward running, followed by 5 min of general stretching of the leg muscles at the comfort of the participant); then, the participants made three maximal vertical squat jumps, where the maximum height that the top of the head reached was taken as the highest point of the jump. Participants were next instructed to aim for a squat jump height corresponding to 95% of the highest point of the jump while maintaining their trunk position and a 90° knee joint angle by placing two markers on the wall for each participant. To do this, the researcher stood on a chair to see whether the head height reached the marker set on the wall, while the co-supervisor sat on a chair to observe whether the trunk position was maintained, and a knee joint angle of 90° was achieved by monitoring the squat phase of the jump at the second marker on the wall. Ninety-five percent of the maximal vertical squat jump head height then served as a target height that the participants were instructed to achieve for each jump. All participant squat jumps had to be executed correctly and where participants were unable to satisfy the target jump height, trunk position, or the 90° knee joint angle targets, they were stopped and given a 1 min rest interval before having to complete that set. The maximal vertical squat jump target heights were adjusted after 2 weeks of training based on the participant’s jump height advances, while the CNT group rested for 8 weeks. The post-jump performance test was conducted three days after the final training session by both the CNT and the TRN group. The study protocol was consistent with protocols used in previous publications [17,19,23], where we showed that acute bouts of this type of training in sedentary individuals result in muscle histological damage and were thus confident that adaptation would result from the training.

### 2.4. Biochemical Parameters

Blood samples were collected from the antecubital vein site in vacuum tubes containing an anticoagulant (0.004% EDTA) PRE (10 days before) and POST (3 days after) the training/rest period. The lactic dehydrogenase (LDH) and creatine kinase (CK) levels were determined using a one-step sandwich assay (Access CK-MB assay, Beckman Coulter, Inc., Chaska, Minnesota).

### 2.5. Functional Test

Three maximal standing vertical jumps were executed by each participant tethered to a spring cable around their waist PRE and POST the training/rest period. With the aid of a FitroDyne (TENDO Weightlifting Analyzer V-206, AMR sport, Queensland, Australia) device [25], the average and peak velocity (m/s) and average and peak power (w) of the study participants’ maximal standing vertical jump heights were recorded. The vertical jump was achieved from a standing still position and was preceded by a quick semi-crouching non-countermovement action with the arms extended backwards and the knees not bent past 90° using the chalk on finger method. Three jump trials were completed with 10–15 s of rest allowed between jump trials and the best score was recorded. The difference between the maximal vertical jump height and the standing reach height with shoes on was documented as the actual maximal vertical jump height.

### 2.6. Muscle Biopsy and Sample Preparation

A medical doctor performed the vacuum-aided needle biopsy technique to obtain muscle biopsies from the midpoint of the vastus lateralis muscle from the randomly chosen dominant or non-dominant leg of each study participant [26]. The instruments used for the procedure were sterilised and available prior to the procedure. For the procedure, participants settled in the supine position with the thigh to be biopsied exposed, while the skin over the biopsy site was prepared with iodopovidone. A local anaesthetic was administered to the skin, subcutaneous tissue and muscles, and after 3–5 min, the biopsy site was evaluated for sensitivity to pain. An incision of 1 cm was made to enhance the penetration of the biopsy needle using a size 15 surgical blade. The biopsy needle was inserted into the body of the muscle, and while the doctor excised the muscle, a trained researcher created suction to draw the muscle into the needle. After the sample was removed, bleeding at the biopsy site was stalled with gentle pressure and the site was covered with a sterile Band-Aid. Muscle biopsies were obtained 10 days prior to and 3 days after either 8 weeks of rest or jump training. These muscle biopsies were then frozen in pre-cooled isopentane in liquid nitrogen after being embedded in an optimal cutting temperature compound (Leica Microsystem Nussloch GmbH, Nussloch, Germany) for subsequent cryo-microtoming and immunofluorescence microscopy. All muscle biopsy samples were stored at −80 °C. 

### 2.7. Immunofluorescence Staining

The muscle tissue was cross-sectioned at −22 °C using a cryo-microtome (Leica CM1100, Leica Microsystem Nussloch GmbH, Nussloch, Germany). The muscle tissue sections were mounted on microscope slides and placed in a freezer (−20 °C) overnight. The next day, the slides were thawed, permeabilised in 0.01 M phosphate-buffered saline (PBS) containing 0.25% Triton X-100 (15 min) and washed with PBS (3 × 5 min). The myofibres were labelled with MHC II (myosin heavy chain II, 1:250; A4.74, mouse monoclonal antibody, Developmental Studies Hybridoma Bank, Iowa City, IA, USA) primary antibody for 1 h, after which positive fibres were observed with an alexa fluor 488 secondary antibody conjugate (1:250, goat anti-mouse, Invitrogen, Eugene, OR, USA) to discern the type II muscle fibres. To observe the sarcolemmas, myofibres were incubated with dystrophin (1:250, rabbit polyclonal, Santa Cruz Biotechnology, Santa Cruz, CA, USA) primary antibody and alexa fluor 594 (1:250, goat anti-rabbit, Invitrogen, Eugene, OR, USA) secondary antibody conjugate for 1 h at room temperature. All sections were labelled with bisBenzimide H 33342 trihydrochloride (1:200, Hoechst, B2261, Merck, Darmstadt, Germany) to visualise the nuclei. The tissue sections were then rinsed in PBS (3 × 5 min) and mounted with a fluorescent mounting medium (DAKO; GLostrup, Denmark). 

### 2.8. Quantitative Image Analysis

**Figure 2:**
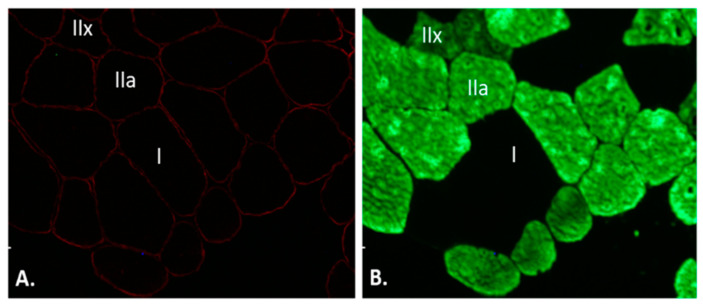
Immunofluorescence of the cross-sectional area of muscle fibres biopsied 10 days before the plyometric jump training. Dual immunofluorescence staining with anti-dystrophin (**A**) and anti-myosin heavy chain II (**B**) was performed. IIx indicates MHC-IIx-positive fibres; IIa indicates MHC-IIa-positive fibres; I indicates MHC-I-positive fibres. Scale bars: 20 um.

The tissue sections were then analysed using a fluorescence microscope (model DM 5000 CTR; Leica Microsystem Nussloch GmbH, Nussloch, Germany) with a 20× objective. The images were evaluated for myofibre positivity to MHC II antibody to determine the fibre type (Figure 2), where type IIa fibres were discerned from type IIx fibres via a greater fluorescent staining intensity [17]. This allowed for the counting of fibre types and quantification of muscle CSA using the ImageJ 1.41 software open-source software [27,28].

### 2.9. Statistical Analysis

An assessment for normality was conducted on the data set by inspecting the normal probability plots. A one-way, mixed-model, repeated-measures analysis of variance was employed to analyse the changes over time in both groups. Further significant differences were observed by evaluating the post hoc Fisher’s least significant difference test results. An α of *p* < 0.05 was accepted as significant.

## 3. Results

### 3.1. Blood Markers of Muscle Damage

The participants of both groups presented low blood marker levels of muscle damage PRE and POST the training/rest period (CK: TRN 128.7 ± 66.9 and 119.3 ± 59.8, respectively; CNT 126.2 ± 21.1 and 154.2 ± 45.2, respectively; LDH: TRN 238.4 ± 93.0 and 157.9 ± 25.4, respectively; CNT 206.4 ± 40.0 and 173.0 ± 31.3, respectively). No significant differences were observed between the groups.

### 3.2. Functional Test Measures and Jump Height Performance

**Figure 3:**
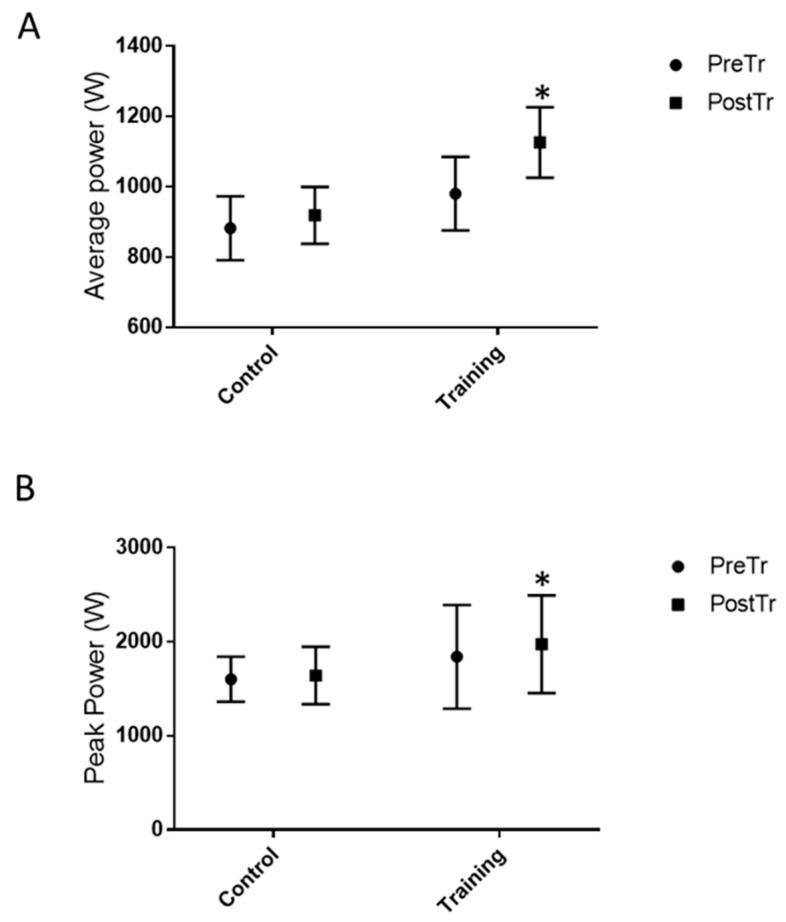
Functional test measures: (**A**) average power (W) and (**B**) peak power (W) before and after 8 weeks of plyometric jump training or rest. Peak values represent the highest of the three test jumps. * *p* < 0.005.

**Figure 4:**
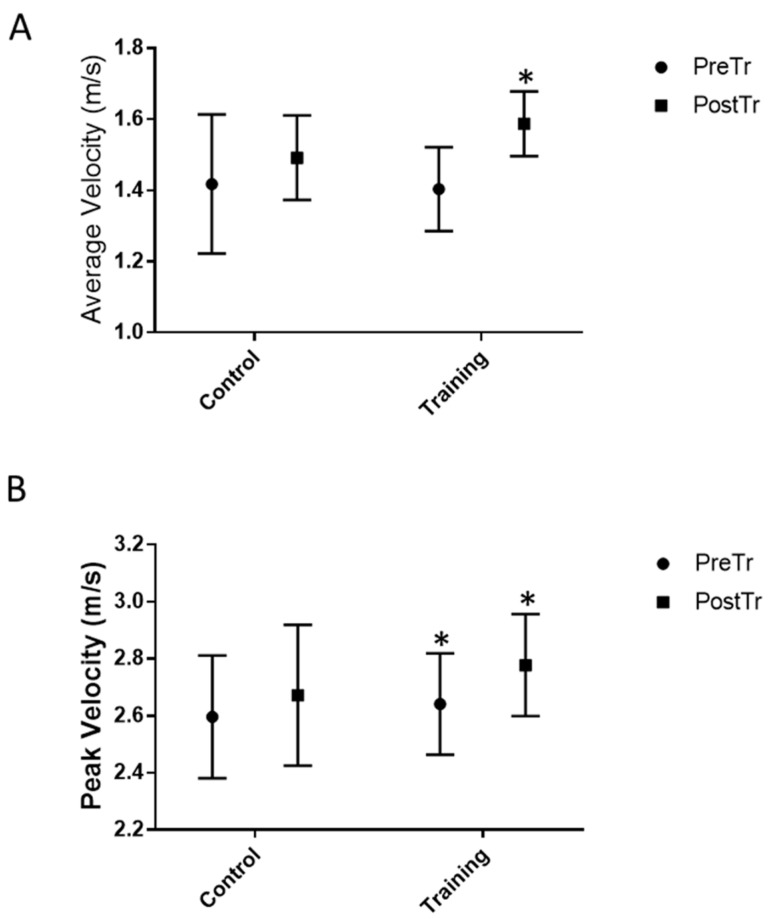
Functional test measures: (**A**) average velocity (m/s) and (**B**) peak velocity (m/s) before and after 8 weeks of plyometric jump training or rest. Peak values represent the highest of the three test jumps. * *p* < 0.005.

**Figure 5:**
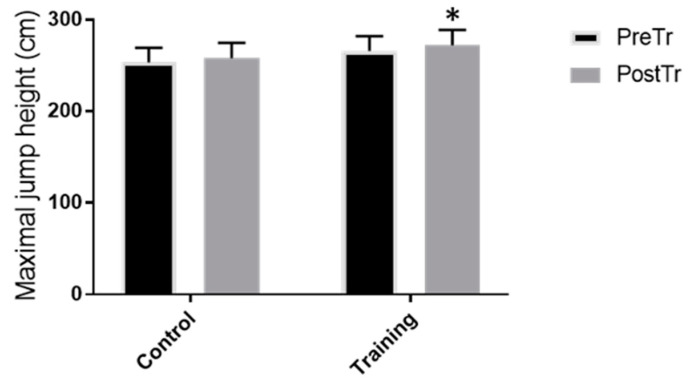
Functional test measures: jump height (cm) before and after 8 weeks of plyometric jump training or rest. Peak values represent the highest of the three test jumps. * *p* < 0.001.

After 8 weeks of jump training, the average and peak power (Figure 3) significantly increased in the TRN group (*p* < 0.001 and *p* < 0.005, respectively; mean increases in average power: 16.7 ± 0.1% and peak power: 8.2 ± 0.1%). The average and peak velocity (Figure 4) increased significantly (*p* < 0.005 and *p* < 0.005, respectively) in the TRN group following jump training (mean increases in average velocity: 13.7 ± 0.1% and peak velocity: 5.2 ± 0.0%). The vertical jump height significantly increased in the TRN group after the plyometric jump training by 25 ± 0.0% (*p* < 0.0003), whereas no significant differences were observed for the CNT group for the functional test measures (data shown in Figure 3 and Figure 4) and jump height performance (data shown in Figure 5). Eight weeks of plyometric squat jump training increased the average and the peak power of the squat jump by 14.8% and 7.3%, respectively, compared with the control group (4.2% and 2.4%, respectively).

### 3.3. Skeletal Muscle Fibres Adaptations

**Figure 6:**
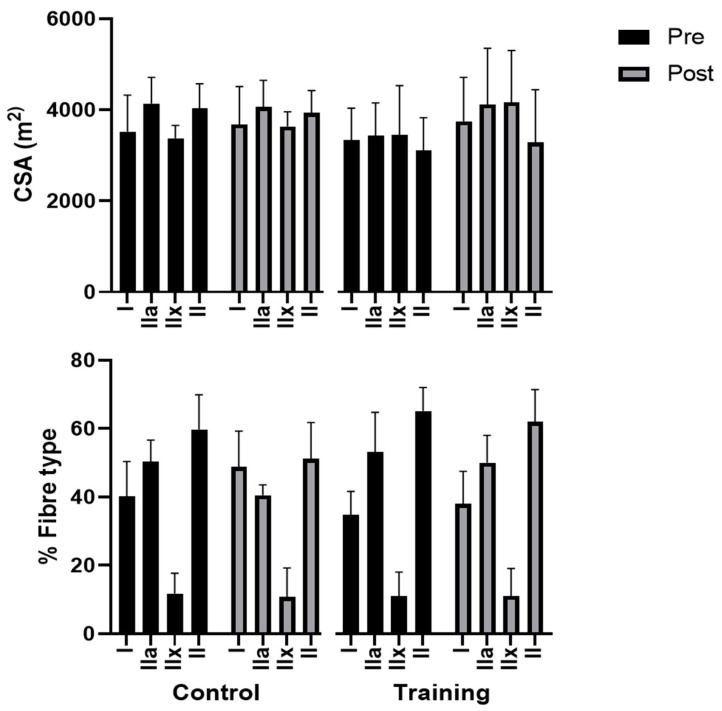
Muscle fibre type cross-sectional area (µm^2^) and percentage (%) of vastus lateralis muscles before and after 8 weeks of plyometric jump training. II indicates MHC-IIx + IIa fibres; IIx indicates MHC-IIx fibres; IIa indicates MHC-IIa fibres; I indicates MHC-I fibres.

No significant differences in the skeletal muscle fibre CSAs between groups and amongst individuals were found. Furthermore, no change in the skeletal muscle fibre percentage ratios were found in either the TRN or CNT groups following 8 weeks of plyometric squat jumping (*p* > 0.07, data shown in Figure 6).

## 4. Discussion

In the present study, the skeletal muscle histological adaptations and the changes in jump performance parameters in response to 8 weeks of low-volume plyometric squat jump training in inactive subjects were investigated. It is important to have a plyometric jumping protocol that can be introduced safely to inactive individuals since the study participants did not take part in any form of physical activity and had no sporting background. It was shown that plyometric squat jumping for individuals with these characteristics poses a particular risk for exertional rhabdomyolysis [19]. In the current study, the safety of the effect of the exercise protocol was assessed by monitoring the markers of muscle damage in the blood to detect a condition known as rhabdomyolysis [8]. Exertional rhabdomyolysis is diagnosed clinically when the CK activity exceeds 1000 U/L [29] and may affect sedentary subjects who take part in unaccustomed exercise for the first time.

At the conclusion of the experimental period, the trained group showed significant improvements in all jump performance parameters assessed but these results did not include skeletal muscle fibre adaptations (CSA or a muscle-fibre-type transition).

The current study showed that 8 weeks of plyometric squat jump training significantly increased the average and peak power of squat jumping compared with the control group. This would allow the trained individuals’ working muscles to produce a greater acceleration for the jump over a shorter period and allow for a greater magnitude of work per unit of time [30]. The gains in velocity and power translated into a significant improvement in maximal vertical jump height in the trained group compared with the control group. This corroborated studies that indicated a strong association between power measures and vertical jump performance [31,32,33], which indicates that power influences vertical jump performance [34], although these studies used a high volume of jumps. Since jump performance ability is dependent on an individual’s skill to capitalise on elastic and neural benefits of the SSC and the power and velocity of the activated muscles during contraction, it is to be expected that plyometric jump training may result in improved jump performance [30]. Markovic, 2007 conducted a meta-analytical approach to determine whether plyometric jump training improves vertical jump height [35]. The study concluded that plyometric training resulted in improved vertical jump height and that various parameters may have influenced the result, including the participant characteristics, training program design and the use of diverse testing protocols. We previously showed that type II muscle fibres are preferentially damaged due to plyometric squat jump exercise [17] and we, therefore, hypothesised that they would be more likely to produce skeletal muscle adaptation, mainly in type IIa and IIx muscle fibres. Moreover, we observed that skeletal muscle damage commences at the ultrastructural level presenting as Z-line streaming, although the sarcomere integrity seems to return to normal following 8 weeks of squat jump training [23].

However, the functional improvements in the current study did not culminate in the adaptation of the muscle characteristics assessed in the current study, including an increased muscle CSA and a muscle fibre percentage distribution change, even between the sub-types of type II fibres. Another contributing factor may have been the large variability in the CSAs and fibre-type percentage distributions between the subjects observed at baseline. Concerning muscle-fibre-type adaptations following plyometric squat jump training, various authors showed that functional measures were improved with 6 weeks [36], 8 weeks [37] and 15 weeks of plyometric training while the fibre-type distribution remained unchanged. The squat jump training protocol employed in the current study could be regarded as a form of training that is beneficial for beginners since it was simple and safe to be executed by inactive individuals. 

A study by Vissing et al. reported significant increases in the maximum countermovement jump height and maximum power during countermovement jumps following 12 weeks of plyometric training [6]. An increased fibre CSA did not accompany these improvements. However, Potteiger et al. reported that 8 weeks of plyometric training increased both the type I and type II muscle fibre CSAs by 4.4% and 7.8%, respectively [9]. Although the training protocol had a similar training period, the training included a variety of jumping activities, such as vertical, bounding and depth jumps, and included more jump repetitions per training session, which may have been the reason for the change in the fibre characteristics observed. However, it is important to note that all the study subjects had regularly performed resistance training and aerobic exercise for at least 3 months prior to the study commencement. Behrens et al. studied knee extensor neuromuscular adaptation in response to 8 weeks of plyometric training [38]. Although hypertrophic changes were not studied, the authors reported functional improvements in squat and countermovement jump heights, which were accompanied by a greater neural drive to the quadriceps muscle, as measured by the isometric maximum voluntary torque, rate of torque development and impulse over various time intervals. 

Recently, Monti et al. evaluated the mean CSA of the vastus lateralis muscle with ultrasonography images taken at different points along the femur length following 6 weeks of plyometric training performed on a novel training device [39]. At the 60% femur length site, similar to where muscle biopsies were obtained in our study, the authors observed an increase in the mean CSA of the whole vastus lateralis muscle following 6 weeks of training, while we did not observe any increase in individual muscle fibre CSAs. The reasons for the differences found in the studies could include the type of jumps performed (since the training device that was used mimicked drop jumps and not squat jumps), the higher number of jump repetitions per training session and the type of technique used to detect muscle hypertrophy. Intramuscular swelling could also be responsible for the findings of the increased muscle CSAs since this goes undetected when using ultrasonography.

Limitations of the current study included the low number of subjects recruited and the specificity of using only one gender and age group in the study population. Due to the invasive method used in the study, the number of participants was limited and the sample size was selected based on a previously published manuscript where we observed histological differences due to plyometric training [23]. The large variation in the body weight and height could account for the variability and, subsequently, the non-significant findings related to the skeletal muscle histological changes observed. It would also be interesting to evaluate histological changes resulting from plyometric training on other muscles of the lower extremity, such as the vastus medialis, rectus femoris, gastrocnemius and triceps surae. Moreover, body fat and nutritional habits were not evaluated in this study, along with kinematic measures and neural adaptation mechanisms. Future studies should therefore include body fat measures, nutritional habits, a higher number of study participants, kinematic measures and an investigation of how neural adaptation affects functional performance. Future studies should also include young adult female study participants and evaluate how age and gender may influence the results obtained. In terms of functional performance markers, we would expect there to be no differences in performance improvements between males and females with plyometric training [40], although skeletal muscle gender dimorphism is observed in the general muscle mass, size of individual fibres, metabolic enzyme activities, hormones and gene expression profiles [41]. This sex difference in muscularity may influence the adaptation of muscle morphological characteristics between males and females. Ageing is associated with a fast-to-slow-fibre-type transition accompanied by a loss of maximum strength, muscle mass and functional deterioration [42]. The current training protocol could potentially be of more value to this population group by greatly improving functional performance and skeletal muscle contractile properties [43,44]. Furthermore, the fibre transition associated with ageing may be attenuated by plyometric training since there is evidence to suggest that a moderate protocol increases muscle mass in this group, as determined using ultrasonography [45].

Investigating histological changes due to specific types of plyometric jumps and increasing the volume of jumps from low to moderate for this subject cohort should include continuous monitoring of markers of muscle damage during the study period to assess the risk for rhabdomyolysis, which may result in skeletal muscle histological adaptations.

## 5. Conclusions

In conclusion, by employing a simple plyometric squat jump training protocol, inactive individuals were able to improve functional measures, including jump velocity and power, resulting in better squat jump height performances following 8 weeks of squat jump training without adaptation in the cross-sectional area of skeletal muscle fibres. The findings of improved functional performance may be more likely explained by the improvements in neural adaptations involving neural recruitment and intermuscular coordination, although we cannot exclude ultrastructural changes that did not directly affect the MHC or the CSA. Further, it can be concluded along with the literature discussed above that this relatively simple protocol employed for the current study is safe for beginners, whilst more complex jumping tasks and a variety of jumping tasks should be undertaken for both performance and muscle fibre size changes. 

## Figures and Tables

**Table 1:** Subject characteristics.

	Control Group (*n* = 5)	Training Group (*n* = 8)	Total (*n* = 13)
Age (year)	21.4 ± 1.7	21.5 ± 1.7	21.5 ± 1.7
Height (cm)	169.3 ± 10.2	176.2 ± 10	173.6 ± 10.3
Weight (kg)	63.0 ± 6.8	71.6 ± 21.8	68.3 ± 17.7
Ave max squat jump height (cm)	215 ± 13.6	224 ± 12.6	220.2 ± 13.7
95% squat jump height (cm)	204 ± 12.9	212 ± 12.0	209.2 ± 13

Data presented as mean ± SD.

## Data Availability

Data supporting reported results can be found at the Stellenbosch University library e-theses collection (https://library.sun.ac.za/en-za/Pages/Home.aspx accessed on 15 November 2021).
